# Improved pregnancy outcomes in women with type 1 and type 2 diabetes but substantial clinic-to-clinic variations: a prospective nationwide study

**DOI:** 10.1007/s00125-017-4314-3

**Published:** 2017-06-08

**Authors:** Helen R. Murphy, Ruth Bell, Cher Cartwright, Paula Curnow, Michael Maresh, Margery Morgan, Catherine Sylvester, Bob Young, Nick Lewis-Barned

**Affiliations:** 1 Norwich Medical School, Floor 2, Bob Champion Research and Education Building, University of East Anglia, Norwich, NR4 7UQ UK; 2 Division of Women’s Health, North Wing, St Thomas’ Campus, Kings College London, London, UK; 3 Institute of Health and Society, Newcastle University, Newcastle upon Tyne, UK; 4 Clinical Audits & Registries Management Service, NHS Digital, Leeds, UK; 5 Department of Obstetrics, St Mary’s Hospital, Central Manchester University Hospital NHS Foundation Trust, Manchester Academic Health Science Centre, Manchester, UK; 6 Department of Obstetrics, Singleton Hospital, Abertawe Bro Morgannwg, Swansea, UK; 7 Department of Diabetes and Endocrinology, Northumbria Healthcare NHS Foundation Trust, Northumberland, UK

**Keywords:** Antenatal, Congenital anomaly, Diabetes, Glucose, Large for gestational age, Neonatal, Pregnancy, Pre-pregnancy care, Preterm, Stillbirth

## Abstract

**Aims/hypothesis:**

The aim of this prospective nationwide study was to examine antenatal pregnancy care and pregnancy outcomes in women with type 1 and type 2 diabetes, and to describe changes since 2002/2003.

**Methods:**

This national population-based cohort included 3036 pregnant women with diabetes from 155 maternity clinics in England and Wales who delivered during 2015. The main outcome measures were maternal glycaemic control, preterm delivery (before 37 weeks), infant large for gestational age (LGA), and rates of congenital anomaly, stillbirth and neonatal death.

**Results:**

Of 3036 women, 1563 (51%) had type 1, 1386 (46%) had type 2 and 87 (3%) had other types of diabetes. The percentage of women achieving HbA_1c_ < 6.5% (48 mmol/mol) in early pregnancy varied greatly between clinics (median [interquartile range] 14.3% [7.7–22.2] for type 1, 37.0% [27.3–46.2] for type 2). The number of infants born preterm (21.7% vs 39.7%) and LGA (23.9% vs 46.4%) were lower for women with type 2 compared with type 1 diabetes (both *p* < 0.001). The prevalence rates for congenital anomaly (46.2/1000 births for type 1, 34.6/1000 births for type 2) and neonatal death (8.1/1000 births for type 1, 11.4/1000 births for type 2) were unchanged since 2002/2003. Stillbirth rates are almost 2.5 times lower than in 2002/2003 (10.7 vs 25.8/1000 births for type 1, *p* = 0.0012; 10.5 vs 29.2/1000 births for type 2, *p* = 0.0091).

**Conclusions/interpretation:**

Stillbirth rates among women with type 1 and type 2 diabetes have decreased since 2002/2003. Rates of preterm delivery and LGA infants are lower in women with type 2 compared with type 1 diabetes. In women with type 1 diabetes, suboptimal glucose control and high rates of perinatal morbidity persist with substantial variations between clinics.

**Data availability:**

Further details of the data collection methodology, individual clinic data and the full audit reports for healthcare professionals and service users are available from http://content.digital.nhs.uk/npid.

## Introduction

Pregnancy in women with diabetes is associated with increased risks of serious adverse outcomes with a two-to-five fold increased risk of congenital anomaly, stillbirth and neonatal death compared with the general maternity population [1–3]. Less severe but more frequent perinatal complications relating to maternal diabetes include preterm delivery, large for gestational age (LGA) infants and neonatal intensive care unit admission, affecting approximately one in two babies [4, 5]. Nationwide studies from the Netherlands, Sweden and Finland suggest no improvement in either rates of serious adverse pregnancy outcomes or perinatal complications in recent decades, with possible explanations including increasing maternal age, longer duration of diabetes and increasing rates of overweight and obesity among women of reproductive years.

A 2002/2003 population-based study, conducted by the Confidential Enquiry into Maternal and Child Health (CEMACH), examined the quality of maternity care and documented pregnancy outcomes in the UK among women with diabetes, concluding that pregnancy preparation was inadequate, resulting in potentially modifiable poor pregnancy outcomes in both type 1 and type 2 diabetes [6]. Since then the National Institute for Health and Care Excellence (NICE) have developed diabetes pregnancy guidelines with a clear emphasis on improving provision of prepregnancy and antenatal diabetes care [7]. The NICE guideline recommendations for prepregnancy preparation include taking 5 mg preconception folic acid, presenting for antenatal care before 8 weeks’ gestation and avoiding potentially harmful medications. The 2015 update lowered the maternal glycaemic control target from HbA_1c_ < 7.0% (53 mmol/mol) to HbA_1c_ < 6.5% (48 mmol/mol) and recommended elective delivery between 37^+0^ and 38^+6^ weeks’ gestation [8].

Responding to the NICE guidelines, a National Pregnancy in Diabetes (NPID) audit was established to document the pregnancy preparation, antenatal care and fetal health outcomes for pregnant women with type 1 and type 2 diabetes [9].

Here we present pregnancy outcomes of women with diabetes who delivered between 1 January 2015 and 31 December 2015. Our aims were to provide contemporary data on the rates of serious adverse pregnancy outcomes (congenital anomaly, stillbirth and neonatal death) and perinatal complications (preterm delivery, LGA and neonatal care admission), and to explore the relationship between maternal deprivation and clinic-to-clinic variations with maternal glycaemic control and folic acid supplementation. We also describe changes since the previous 2002/2003 CEMACH survey.

## Methods

Healthcare professionals at each maternity unit completed standardised web-based data entry forms for every pregnant woman with diabetes who delivered between 1 January and 31 December 2015. As not all pregnancies resulted in a delivery these numbers included pregnancies ending in miscarriage or termination within this date range. All maternity units in England, Wales and the Isle of Man providing antenatal diabetes care were expected to participate (http://digital.nhs.uk/npid). Data were obtained only from women who provided written informed consent. The information leaflet and consent form met the Health Research Authority requirements for clinical audit, and research ethics approval was not required. Linkage with data collected in other systems (Hospital Episodes Statistics data, Patient Episode Database for Wales, National Diabetes Audit) allowed only a limited number of data items to be collected by local teams. This included information regarding type of diabetes, medications and folic acid use before conception. Data regarding pregnancy outcome was completed locally at 28 days after delivery, miscarriage or termination.

We defined pregestational diabetes as diabetes that had been diagnosed before pregnancy, and excluded women with diabetes who presented during pregnancy. Women with monogenic diabetes, or where there was doubt about whether they had type 1 or type 2 diabetes, or the type of diabetes data item was missing were classified as ‘other’ (these pregnancies were excluded from analyses comparing type 1 and type 2 diabetes). Glycaemic control was derived from HbA_1c_ measurements, and the first and last recorded values during pregnancy were collected. HbA_1c_ was measured locally using DCCT-aligned assays. We explored the relationship between maternal deprivation with glycaemic control and folic acid supplementation using an index of multiple deprivation score for women living in England and Wales where postcode details were recorded in the National Diabetes Audit [10]. For data protection of potentially sensitive information in the clinic-to-clinic comparisons, we included only clinics with at least ten completed pregnancy records; therefore, 130 clinics were included for type 1 diabetes and 103 clinics for type 2 diabetes comparisons.

We defined stillbirth as a fetal loss occurring after 24 weeks’ gestation, and neonatal death as death of a live born infant up to 28 days after delivery. We collected data on congenital anomalies for live births and terminations of pregnancy at any gestation, including for stillbirths and for fetal loss after 20 weeks’ gestation. The reported diagnoses for congenital anomaly were obtained from the hospital ICD-10 codes (www.who.int/classifications/icd/en/). We calculated the congenital anomaly rate as the number of infants with one or more congenital anomalies divided by the number of live births, terminations, stillbirths and fetal loss after 20 weeks’ gestation. Infant birthweight was adjusted for maternal ethnicity, height and weight, infant sex, and gestational age at delivery for singleton pregnancies using customised centiles with large and extreme LGA defined as >90th and >97.7th percentiles, respectively (GROW Centile Calculator v5.7.7.1, Gestation Network, www.gestation.net) [11].

Variables which were not normally distributed are given as median (interquartile range [IQR]), while normally distributed variables are given as mean (SD). Univariate analyses comparing the proportions between groups were performed using *z* tests and *t* tests for comparing continuous variables. We used Stata 8.0 for analyses and Poisson distribution to obtain 95% CIs for the rate and prevalence ratios.

## Results

### Participation

During the 12 month study period, 155 National Health Service (NHS) maternity clinics participated. Together they contributed 3044 pregnancies among 3036 women with diabetes, providing data for 3086 pregnancies (eight women had two pregnancies and 42 twin pregnancies were recorded). We report infant health outcomes for 2866 pregnancies that continued beyond 24 weeks’ gestation. Table 1 gives a description of the numbers of women and pregnancies according to type of diabetes.

**Table 1:** Numbers of women, pregnancies and infants registered in the study during 2015

	All	Type 1 diabetes	Type 2 diabetes	Other
Women	3036	1563	1386	87
Pregnancies	3044	1566	1391	87
Total pregnancy outcomes	3086^a^	1587	1409	90
Pregnancies ongoing after 24 weeks	2866	1470	1313	83
Live births after 24 weeks	2868	1474	1313	81
Stillbirth	35	16	14	5
Infants born after 24 weeks	2903	1490	1327	86
Infants with unknown gestation	4	1	3	6
Live births before 24 weeks	1	1	0	0
Total registered births	2908	1492	1330	86

^a^Eight women had two pregnancies and 42 twin pregnancies were recorded among 3036 women, providing outcome data for 3086 pregnancies

### Maternal characteristics

Almost half of the women included in the study (*n* = 1386; 46%) had type 2 diabetes, rising to more than 50% in large metropolitan regions (London, West Midlands, Yorkshire and Humber). Ninety per cent of Asian women and 71% of Black women had type 2 diabetes. As expected, women with type 2 diabetes were significantly older (33.6 vs 29.9 years; *p* < 0.001), had higher BMI (33.3 vs 26.8 kg/m^2^; *p* < 0.001), had shorter diabetes duration (4.8 vs 14.9 years; *p* < 0.001) and were more likely to live in the most deprived quintile of deprivation (38.5% vs 20.9%; *p* < 0.001) than women with type 1 diabetes (Table 2).

**Table 2:** Maternal and neonatal characteristics by diabetes type

	Type 1 diabetes *n* = 1563 (53%)^a^	Type 2 diabetes *n* = 1386 (47%)^a^	*p* value
Age at delivery (years)	29.9 (5.7)	33.6 (5.2)	<0.001
Duration of diabetes (years)	14.9 (8.3)	4.8 (4.3)	<0.001
BMI (kg/m^2^)^b^	26.8 (5.6)	33.3 (7.3)	<0.001
BMI category			
18.5–24.9	660 (42%)	159 (11%)	
25–29.9	473 (30%)	264 (19%)	
≥ 30	335 (21%)	885 (64%)	
Ethnicity	*n* = 1201	*n* = 884	
White	883 (74%)	406 (46%)	<0.05
Asian	27 (2%)	247 (28%)	
Black	32 (3%)	79 (9%)	
Mixed/other	43 (4%)	47 (5%)	
Not stated/unknown	216 (18%)	105 (12%)	
Deprivation quintile	*n* = 1197	*n* = 880	<0.001
1: least deprived	17.5%	7.4%	
2	18.6%	11.5%	
3	21.7%	17.0%	
4	21.3%	25.6%	
5: most deprived	20.9%	38.5%	
5 mg preconception folic acid	720 (46.1%)	312 (22.5%)	<0.001
Booking before 8 weeks	850 (54.4%)	501 (36.2%)	<0.001
Potentially harmful medications	45 (2.9%)	119 (8.6%)	<0.001
Early pregnancy HbA_1c_	*n* = 1306	*n* = 1024	
%	7.6 (6.8–8.7)	6.8 (6.2–8.0)	<0.001
mmol/mol	60.0 (51.0–72.0)	51.5 (44.0–64.3)	
HbA_1c_ < 6.5% (48 mmol/mol)	16.2%	38.3%	
Late pregnancy HbA_1c_	*n* = 1210	*n* = 1017	
%	6.7 (6.1–7.5)	5.9 (5.5–6.5)	<0.001
mmol/mol	50 (43–58)	41 (37–47)	
HbA_1c_ < 6.5% (48 mmol/mol)	40.0%	76.0%	
Perinatal outcomes^c,d^			
Gestational age at delivery (weeks)	36.4 (2.0)	37.1 (2.0)	<0.001
Preterm delivery <37^+0^ weeks	568 (39.7%)	278 (21.7%)	<0.05
LGA >90th percentile	667 (46.4%)	307 (23.9%)	<0.05
LGA >97.7th percentile	423 (29.4%)	180 (14.0%)	
Serious adverse pregnancy outcome^d^			
Congenital anomaly	69 (46.2/1000)	46 (34.6/1000)	NS
Stillbirth	16 (10.7/1000)	14 (10.5/1000)	NS
Neonatal death	12 (8.1/1000)	15 (11.4/1000)	NS

Data are presented as *n* (%), mean (SD), median (IQR) or *n* (*n* per 1000 births)

^a^87 (3%) women had ‘other’ types of diabetes, these pregnancies were excluded from analyses comparing type 1 and type 2 diabetes

^b^The maternal BMI at booking was unknown for four women with type 1 and two women with type 2 diabetes

^c^The gestation age at delivery was available for 1433 infants of mothers with type 1 diabetes and for 1280 with type 2 diabetes. The customised birthweight percentiles were calculated for 1438 infants of mothers with type 1 diabetes and for 1287 with type 2 diabetes

^d^The data presented for serious adverse pregnancy outcomes and perinatal complications include only singleton infants

### Prepregnancy preparation

#### Preconception folic acid

**Fig. 1:**
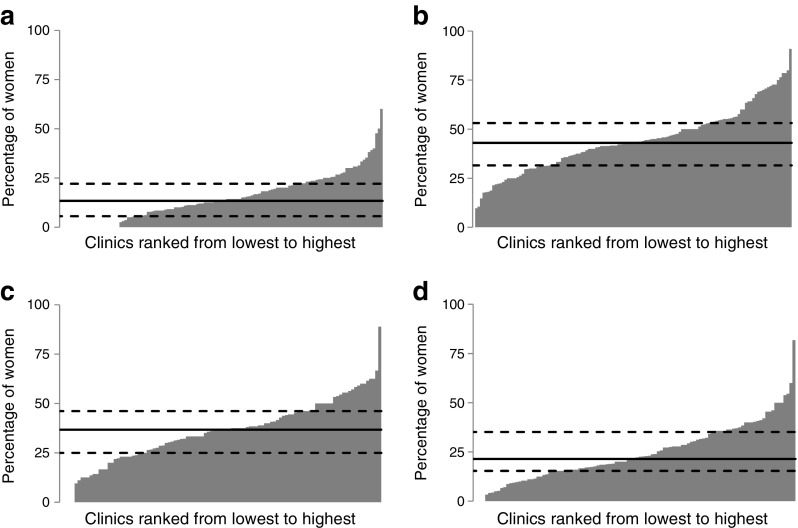
Variation between clinics in the percentage of women taking 5 mg folic acid and achieving target HbA_1c_ levels <6.5% (48 mmol/mol) in early pregnancy. Percentage of women with type 1 diabetes: (**a**) achieving target HbA_1c_ levels in early pregnancy and (**b**) taking 5 mg folic acid and at individual clinics. Percentage of women with type 2 diabetes: (**c**) achieving target HbA_1c_ levels in early pregnancy and (**d**) taking 5 mg folic acid at individual clinics. The clinics are ranked from smallest number to greatest number of women achieving the targets left to right. Solid line, median; dashed lines, IQR

Women with type 2 diabetes were significantly less likely to take the recommended 5 mg dose of folic acid before conception compared with women with type 1 (22.5% vs 46.1%; *p* < 0.001; Table 2). There was also marked variation between clinics in the percentage of women with type 2 diabetes taking 5 mg folic acid (Fig. 1d); 33% of women attending the top quartile clinics took 5 mg folic acid compared with 15% in the lowest quartile (21.3% [15.4–33.2]). Over half the women with type 1 diabetes attending the top quartile clinics took 5 mg folic acid compared with less than a third in the lowest quartile clinics (42.9% [31.5–52.3]; Fig. 1b). Maternal deprivation was strongly associated with folic acid use among women with type 1 diabetes, with 75.1% use among women with type 1 diabetes living in the least deprived areas (vs 37.5% in the most deprived areas; *p* < 0.05, data not shown).

### Glycaemic control in early pregnancy

Although glycaemic control was suboptimal in both type 1 and type 2 diabetes, women with type 2 diabetes were more than twice as likely to achieve target HbA_1c_ levels of <6.5% (48 mmol/mol) in early pregnancy (38.3% vs 16.2%; *p* < 0.001; Table 2). Women achieving target HbA_1c_ levels had lower BMI at booking (25.7 vs 27.0 kg/m^2^ for type 1, 31.9 vs 34.0 kg/m^2^ for type 2; *p* < 0.001). Women with type 1 diabetes who achieved target HbA_1c_ levels were older (31.3 vs 29.8 years; *p* < 0.001) and lived in the least deprived areas, with almost one in four women in the least deprived areas achieving target HbA_1c_ compared with one in ten women in the most deprived areas (24% vs 9.9%; *p* < 0.001). For women with type 2 diabetes, only maternal BMI (31.9 vs 34.0) and shorter diabetes duration (4.2 vs 5.4 years; both *p* < 0.001) were associated with achieving target HbA_1c_ levels (data not shown). The percentage of women with type 1 and type 2 diabetes achieving target HbA_1c_ levels in early pregnancy also varied greatly between clinics (14.3% [7.7–22.2] for type 1 and 37.0% [27.3–46.2] for type 2; Fig. 1a, c). The top quartile clinics had 22% of women with type 1 and 46% with type 2 diabetes achieving target HbA_1c_ levels with 7% and 27%, respectively, in the lowest quartile clinics.

Presenting for antenatal care prior to 8 weeks’ gestation was more common in women with type 1 diabetes (54.4% vs 36.2%; *p* < 0.001; Table 2), again with substantial variation between clinics (55.0% [37.2–65.0] and 36.4% [21.7–50.0], respectively). Periconception exposure to potentially harmful medications, such as statins, ACE inhibitors or angiotensin receptor blockers, was less common in women with type 1 compared with type 2 diabetes (2.9% vs 8.6%; *p* < 0.001).

### Glycaemic control in late pregnancy

As expected, maternal HbA_1c_ levels decreased during pregnancy. After 24 weeks’ gestation in women with type 2 diabetes, 76.0% achieved target HbA_1c_ levels <6.5% (48 mmol/mol) (Table 2) and 85.9% achieved HbA_1c_ < 7.0% (53 mmol/mol) (Table 3). Only 40.0% and 59.5% of women with type 1 diabetes achieved these targets, respectively (*p* < 0.001).

**Table 3:** Comparisons between the NPID 2015 and CEMACH 2002/2003 cohorts

	NPID 2015	CEMACH 2002/2003
Number of women	3036	2359
Number of infants	2866	2400
Number of clinics^a^	155	231
Pregnancies per clinic	19.6	10.2
Type 1 diabetes^b^	1563 (51%)	1707 (72%)
Type 2 diabetes^b^	1386 (46%)	652 (28%)
Maternal duration of diabetes (years)		
Type 1 diabetes	14.9	13
Type 2 diabetes	4.8	3
Preconception folic acid (any dose)^c^		
Type 1 diabetes	51.7%	42.9%
Type 2 diabetes	33.7%	29.4%
Maternal glycaemic control		
Early pregnancy measurement <13 weeks	78%	67%
Type 1 diabetes		
HbA_1c_ %	7.6 (6.8–8.7)	7.5 (6.6–8.5)
HbA_1c_ mmol/mol	60 (51–72)	58 (48–69)
HbA_1c_ < 7% (53 mmol/mol)	29.4%	35.2%
Type 2 diabetes		
HbA_1c_ %	6.8 (6.2–8.0)	7.0 (6.1–8.1)
HbA_1c_ mmol/mol	51 (44–64)	53 (43–65)
HbA_1c_ < 7% (53 mmol/mol)	52.4%	49.0%
Late pregnancy >24 weeks		
Type 1 diabetes		
HbA_1c_ %	6.7 (6.1–7.5)	6.6 (6.0–7.3)
HbA_1c_ mmol/mol	50 (43–58)	49 (42–56)
HbA_1c_ < 7% (53 mmol/mol)	59.5%	65.0%
Type 2 diabetes		
HbA_1c_ %	5.9 (5.5–6.5)	6.3 (5.7–6.9)
HbA_1c_ mmol/mol	41 (37–47)	45 (39–52)
HbA_1c_ < 7% (53 mmol/mol)^d^	85.9%	75.4%
Severe adverse pregnancy outcomes		
Congenital anomaly^e^	117	109
Type 1 diabetes	69 (46.2/1000)	81 (48/1000)
Type 2 diabetes	46 (34.6/1000)	28 (43/1000)
Stillbirth	30	63
Type 1 diabetes	16 (10.7/1000)	44 (25.8/1000)
Type 2 diabetes	14 (10.5/1000)	19 (29.2/1000)
Neonatal death	27	22
Type 1 diabetes	12 (8.1/1000)	16 (9.6/1000)
Type 2 diabetes	15 (11.4/1000)	6 (9.5/1000)

Data are presented as *n* (%), median (IQR) or *n* (*n* per 1000 births)

^a^CEMACH included clinics from England, Wales and Northern Ireland, and informed consent was not obtained. NDIP 2015 included clinics from England, Wales and the Isle of Man, and only collected data with written informed consent

^b^87 (3%) women with ‘other’ types of diabetes are included for comparison with CEMACH but were excluded from subsequent analyses comparing type 1 and type 2 diabetes

^c^The proportion of women taking preconception folic acid (any dose) has increased significantly in type 1 (*p* < 0.001) and type 2 (*p* = 0.022) diabetes since 2002/2003

^d^The proportion of women with type 2 diabetes achieving HbA_1c_ < 7% (53 mmol/mol) after 24 weeks has increased significantly since 2002/2003 (*p* = 0.05)

^e^CEMACH included only major congenital anomaly, with 141 anomalies among 109 infants. NPID 2015 included major and minor anomalies identified based on the ICD-10 codes for congenital anomalies prior to hospital discharge. There were 156 anomalies among 117 infants (69 for women with type 1, 46 for women with type 2 and two for women with ‘other’ forms of diabetes)

### Perinatal morbidity

#### Preterm delivery

**Fig. 2:**
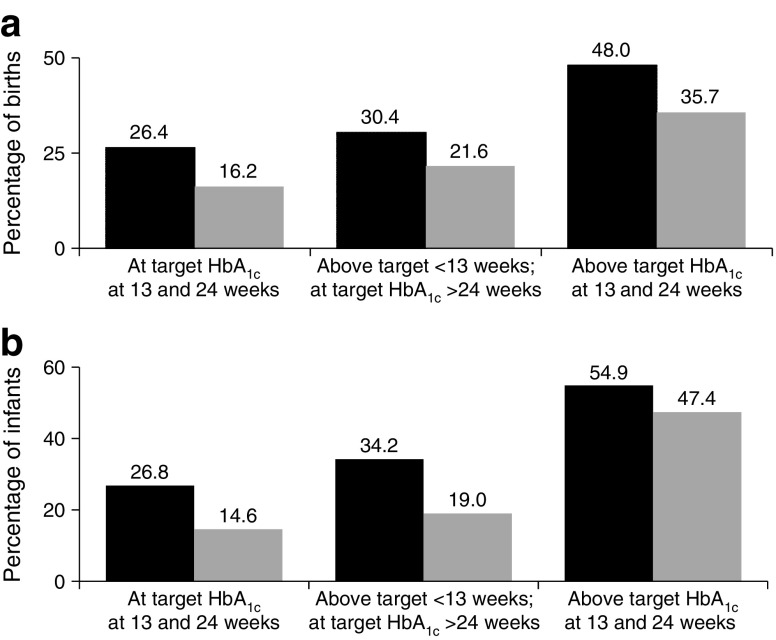
Relationships for achievement of glycaemic control targets (HbA_1c_ < 6.5% [48 mmol/mol]) with (**a**) preterm delivery before 37 weeks’ gestation and (**b**) rates of LGA in infants (customised birthweight >90th percentile). Black bars, type 1 diabetes; grey bars, type 2 diabetes

While the majority of live singleton births were between 37^+0^ and 38^+6^ weeks (mean gestation 36.4 for type 1 and 37.1 for type 2; *p* < 0.001), the rates for preterm delivery before 37 weeks were 39.7% in women with type 1 and 21.7% in those with type 2 diabetes (*p* < 0.05; Table 2). Among women with suboptimal early glycaemic control (HbA_1c_ level ≥ 6.5% [48 mmol/mol]) the preterm birth rate was significantly lower if they later achieved HbA_1c_ levels <6.5% (48 mmol/mol) after 24 weeks’ gestation (30.4% vs 48.0% for type 1; 21.6% vs 35.7% for type 2, both *p* < 0.001; Fig. 2a).

#### LGA

The numbers of large and extreme LGA infants (birthweight >90th and >97.7th percentiles, respectively) were significantly higher for women with type 1 diabetes (46.4% and 29.4% for type 1 vs 23.9% and 14.0% for type 2, respectively; both *p* < 0.001; Table 2). As with preterm delivery, the LGA rates were significantly lower among women with suboptimal early glucose control if they later achieved HbA_1c_ levels <6.5% (48 mmol/mol) (34.2% vs 54.9% for type 1; 19.0% vs 47.4% for type 2, both *p* < 0.001; Fig. 2b).

#### Neonatal intensive care unit admission

LGA infants were more likely to be admitted for neonatal care than infants appropriate for gestational age (32.1% vs 25.6% for type 1, 25.6% vs 11.4% for type 2; *p* < 0.05). Admission to neonatal care units was reduced among term infants (i.e. born at or after 37^+0^ weeks’ gestation) of women with suboptimal glycaemic control in early pregnancy if they later achieved HbA_1c_ levels <6.5% (48 mmol/mol) (22.7% vs 33.4% for type 1 and 12.2% vs 23.6% for type 2; *p* < 0.05).

### Comparisons with the 2002/2003 CEMACH data

#### Maternal and demographic characteristics

This cohort was larger than the previous CEMACH survey, with 685 more diabetes pregnancies and a striking increase in the proportion of pregnancies complicated by type 2 diabetes (Table 3). There was also more concentrated data focusing on pregnancy care across fewer clinics, with 3044 diabetes pregnancies across 155 maternity clinics (19.6 per clinic) in 2015, compared with 2359 diabetes pregnancies in 231 clinics (10.2 per clinic) in 2002/2003. The duration of diabetes was longer (by approximately 2 years) in both type 1 and type 2 diabetes. More women were taking folic acid supplementation before conception and more had a recorded first trimester measurement of HbA_1c_. There was no improvement in glucose control in type 1 diabetes but some improvement during late gestation in type 2 diabetes, with an increased proportion of women who achieved HbA_1c_ < 7.0% (53 mmol/mol) after 24 weeks in 2015 (85.9% vs 75.4%; *p* < 0.05).

#### Serious adverse pregnancy outcomes

**Fig. 3:**
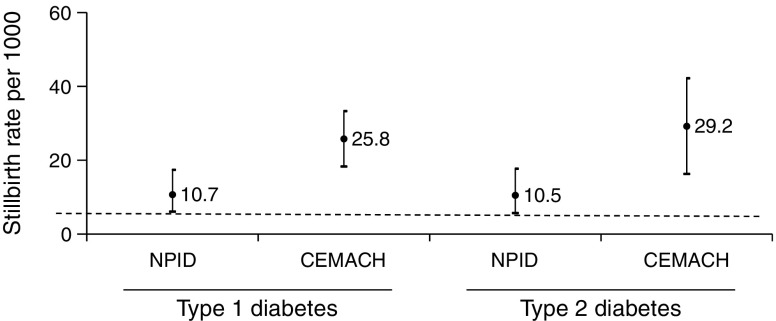
Stillbirth rate during the NPID audit 2015 compared with CEMACH 2002/2003 for women with type 1 and type 2 diabetes. Data presented are stillbirth rates per 1000 births with 95% CI. Dashed line, stillbirth rate for the general maternity population for 2015 (based on data from the Office for National Statistics [12])

In 2015, the stillbirth rate was high (10.7/1000 for type 1 and 10.5/1000 for type 2; Table 3) compared with the general maternity population rate of 4.7/1000 [12]. However, it was almost 2.5-fold lower than in 2002/2003 (Fig. 3). The neonatal death rate was also high (8.1/1000 for type 1 and 11.4/1000 for type 2 diabetes), but unchanged compared with 2002/2003. The prevalence of congenital anomalies (including all major and minor anomalies detected at hospital discharge) was 46.2/1000 for type 1 and 34.6/1000 for type 2 diabetes (Table 3). These data are not directly comparable with the 2002/2003 data, which included only major congenital anomalies.

## Discussion

This large national study details the pregnancy outcomes of women with diabetes more than a decade after the 2002/2003 CEMACH survey. While the pregnancy outcomes of women with diabetes were still suboptimal compared with the general maternity population, substantial progress has been made with significant reductions in stillbirths in both type 1 and type 2 diabetes. There were fewer preterm deliveries, LGA infants and neonatal care unit admissions in women with type 2 compared with type 1 diabetes, reflecting the better antenatal glycaemic control in type 2 diabetes. Glucose control in women with type 1 diabetes was suboptimal, with persistently high rates of preterm delivery, LGA and neonatal care unit admissions. The highest rates of neonatal complications were seen in women with HbA_1c_ levels ≥6.5% (48 mmol/mol) after 24 weeks’ gestation.

Over the past decade the proportion of pregnant women with type 2 diabetes has risen from 28% to 46% of all diabetes pregnancies, with type 2 now outnumbering type 1 diabetes pregnancies in some metropolitan areas. The proportion of women taking preconception folic acid supplementation has increased both in type 1 and type 2 diabetes, which is in contrast to some recent data suggesting a small decline (from 35% to 31%) in the general maternity population [13].

Our data demonstrate the ongoing healthcare inequalities between women with type 1 and type 2 diabetes and the striking contribution of maternal deprivation in type 1 diabetes. In women with type 1 diabetes, 75% of those living in the most socioeconomically advantaged areas took 5 mg preconception folic acid and 25% achieved target HbA_1c_ levels in early pregnancy, whereas only 37% of women living in the most disadvantaged areas took folic acid and only 10% achieved target HbA_1c_ levels in early pregnancy. This information highlights the need to more effectively target prepregnancy care to women living in deprived areas. Previous studies have shown that targeting all women of reproductive years can minimise the impact of maternal deprivation on prepregnancy care attendance, folic acid supplementation and early pregnancy glycaemic control [14].

It is disappointing that so few women with type 1 diabetes achieved the NICE recommended glycaemic control target for HbA_1c_ in early and late pregnancy (16.1% and 40.0% respectively). We speculate that this is due to previously described physiological and pharmacological challenges of matching pre-meal insulin boluses to postprandial glucose profiles in early and late gestation [15, 16]. We have shown that while women achieving target glycaemic control are older, leaner and more socially advantaged, there is substantial variation across clinics, suggesting that the impact of clinic context is also important. Further work is needed to understand whether these clinic-to-clinic variations relate to clinic size and/or staff experience of implementing newer technologies such as insulin pump therapy and continuous glucose monitoring before and during pregnancy.

While there is much emphasis on improving preconception and first trimester glucose control to reduce congenital anomaly, stillbirth and neonatal death, our data also indicate the importance of optimal glucose control in late gestation for reducing the rates of preterm delivery, LGA infants and neonatal intensive care unit admission. An important take home message for women with unplanned pregnancy and/or suboptimal early glucose control is that achieving HbA_1c_ < 6.5% (48 mmol/mol) after 24 weeks was very effective for reducing perinatal complications both in type 1 and type 2 diabetes.

The absolute risk of stillbirth has consistently remained 10–13/1000 over three diabetes pregnancy audit years, which now includes 6675 diabetes pregnancies [17]. Recent data from Sweden have comparable absolute stillbirth rates (15/1000) among women with type 1 diabetes [1]. However, the Swedish diabetes rates are still five times higher than the low background maternity population rates (3/1000) characteristic of high-income countries. In 2002/2003 there was a similar fivefold increase in stillbirths among women with diabetes in the UK [3].

The reductions in stillbirths in type 1 and type 2 diabetes are not solely due to improvements in the general maternity population [12], as the UK currently reports one of the slowest rates of decline in stillbirth of approximately 17% (from 5.3 to 4.4/1000 births) over the past decade [18]. Although there are clear maternal risk factors, including previous stillbirth, multiple pregnancy, nulliparity, diabetes, maternal age over 40 years, non-White ethnicity, smoking and obesity, most of the variability in stillbirth rates is independent of established risk factors [19, 20]. We can only speculate as to the possible explanations for recent improvements, which may include earlier elective birth recommendations (37^+0^ weeks’ gestation), tighter glycaemic control targets (HbA_1c_ < 6.5% or 48 mmol/mol) and/or greater concentrations of women with diabetes among fewer maternity clinics.

Stillbirth is the most common cause of perinatal death [21], so it is surprising that the reduction in stillbirths was not accompanied by a reduction in the neonatal death rate (8.1 vs 9.6/1000 for type 1 and 11.4 vs 9.5/1000 for type 2) since 2002/2003. The reasons for this are unknown and will require further evaluation in larger datasets over longer time frames.

Our study is one of the largest to describe the pregnancy outcomes associated with contemporary diabetes care and includes over 3000 women with pregnancies complicated by diabetes. Our large sample size provides the statistical power for evaluating trends in serious but rare outcomes. Other large-scale studies conducted over 10–20 years in Sweden [1] and Finland [5] are less relevant to current clinical practice, and in particular to the increasing problem of type 2 diabetes in pregnancy [22]. Our cohort is larger than any previous studies of pregnant women with type 2 diabetes and confirms meta-analyses findings that there are no differences in the rates of congenital anomalies, stillbirths and perinatal deaths between type 1 and type 2 diabetes [23]. We do confirm important differences in the perinatal outcomes of type 1 and type 2 diabetes pregnancy with significantly lower rates of preterm delivery, LGA infants and neonatal intensive care unit admissions in type 2 diabetes.

Our study should be interpreted in the context of potential limitations. First, these are cross-sectional analyses, which preclude us from making causal inferences. Second, use of routinely collected data means we have little control over errors during data collection and variations due to differences in timing and laboratory methods for HbA_1c_ levels. Third, because of the information governance requirements and the pre-specified nature of these analyses, we are unable to analyse whether there are significant, independent effects of HbA_1c_ in early and late pregnancy and/or maternal BMI on neonatal outcomes. Furthermore, we cannot guarantee that data from all women and all maternity clinics are included, and we lack information for women who did not provide consent. The numbers of women who refuse consent is anecdotally very small. For 94 clinics that responded to an electronic survey, an average of one woman per clinic was unable to and/or refused consent (C. Cartwright, P. Curnow, C. Sylvester, B. Young, unpublished data). Having 3044 pregnancies in women providing consent is at least comparable to the 2359 pregnancies without consent in 2002/2003. We also cannot be certain that the 87 women where the type of diabetes is classified as ‘unknown’ did not have type 1 or type 2 diabetes.

More work is needed to ensure that women with type 2 diabetes and their community healthcare providers are aware of the importance of safe effective contraception and/or prepregnancy care. Further research is needed to better understand the impact of clinic size and whether centralisation in fewer, larger clinics would improve glucose control and pregnancy outcomes. It remains to be seen whether recent advances in continuous glucose monitoring and closed-loop insulin delivery will be effective for improving late gestation glucose control and reducing perinatal morbidity in type 1 diabetes pregnancy [24, 25]. For stillbirth, research to identify better predictors of placental dysfunction such as erythropoietin, pregnancy associated plasma protein A, alpha fetoprotein and angiogenic/antiangiogenic factors in women with diabetes is needed [26–28].

The NPID data provides information that can be used at the level of individual maternity clinics so service users, healthcare professionals and funders can make informed healthcare choices. It has demonstrated substantial nationwide progress in diabetes stillbirth reductions but highlights ongoing challenges to consistently improve glucose control and reduce perinatal complications in type 1 diabetes pregnancy.

## Abbreviations

CEMACH: Confidential Enquiry into Maternal and Child Health
GROW: Gestation-related optimal weight
IQR: Interquartile range
LGA: Large for gestational age
NHS: National Health Service
NPID: National Pregnancy in Diabetes
NICE: National Institute for Health and Care Excellence
